# Integrin/TGF-β1 Inhibitor GLPG-0187 Blocks SARS-CoV-2 Delta and Omicron Pseudovirus Infection of Airway Epithelial Cells In Vitro, Which Could Attenuate Disease Severity

**DOI:** 10.3390/ph15050618

**Published:** 2022-05-17

**Authors:** Kelsey E. Huntington, Lindsey Carlsen, Eui-Young So, Matthias Piesche, Olin Liang, Wafik S. El-Deiry

**Affiliations:** 1 Laboratory of Translational Oncology and Experimental Cancer Therapeutics, Warren Alpert Medical School, Brown University, Providence, RI 02903, USA; kelsey_huntington@brown.edu (K.E.H.); lindsey_carlsen@brown.edu (L.C.); 2 Department of Pathology and Laboratory Medicine, Warren Alpert Medical School, Brown University, Providence, RI 02903, USA; 3 Joint Program in Cancer Biology, Lifespan Health System and Brown University, Providence, RI 02903, USA; eui_young_so@brown.edu (E.-Y.S.); olin_liang@brown.edu (O.L.); 4 Legorreta Cancer Center, Brown University, Providence, RI 02903, USA; 5 Pathobiology Graduate Program, Warren Alpert Medical School, Brown University, Providence, RI 02903, USA; 6 Hematology-Oncology Division, Department of Medicine, Lifespan Health System and Warren Alpert Medical School, Brown University, Providence, RI 02912, USA; 7 Biomedical Research Laboratories, Medicine Faculty, Universidad Católica del Maule, Talca 3466706, Chile; 8 Oncology Center, Medicine Faculty, Universidad Católica del Maule, Talca 3466706, Chile

**Keywords:** HSAE, GLPG-0187, integrin, MEKi, SARS-CoV-2, TGF-β1, ACE2, COVID-19, Omicron, Delta

## Abstract

As COVID-19 continues to pose major risk for vulnerable populations, including the elderly, immunocompromised, patients with cancer, and those with contraindications to vaccination, novel treatment strategies are urgently needed. SARS-CoV-2 infects target cells via RGD-binding integrins, either independently or as a co-receptor with surface receptor angiotensin-converting enzyme 2 (ACE2). We used pan-integrin inhibitor GLPG-0187 to demonstrate the blockade of SARS-CoV-2 pseudovirus infection of target cells. Omicron pseudovirus infected normal human small airway epithelial (HSAE) cells significantly less than D614G or Delta variant pseudovirus, and GLPG-0187 effectively blocked SARS-CoV-2 pseudovirus infection in a dose-dependent manner across multiple viral variants. GLPG-0187 inhibited Omicron and Delta pseudovirus infection of HSAE cells more significantly than other variants. Pre-treatment of HSAE cells with MEK inhibitor (MEKi) VS-6766 enhanced the inhibition of pseudovirus infection by GLPG-0187. Because integrins activate transforming growth factor beta (TGF-β) signaling, we compared the plasma levels of active and total TGF-β in COVID-19+ patients. The plasma TGF-β1 levels correlated with age, race, and number of medications upon presentation with COVID-19, but not with sex. Total plasma TGF-β1 levels correlated with activated TGF-β1 levels. Moreover, the inhibition of integrin signaling prevents SARS-CoV-2 Delta and Omicron pseudovirus infectivity, and it may mitigate COVID-19 severity through decreased TGF-β1 activation. This therapeutic strategy may be further explored through clinical testing in vulnerable and unvaccinated populations.

## 1. Introduction

Although several highly effective vaccines have now been developed against coronavirus disease 2019 (COVID-19), its threat to public health persists due to the presence of breakthrough cases, the current improbability of achieving herd immunity, reluctance to vaccinate among significant segments of the population, less available vaccines in much of the developing world, and the emergence of highly transmissible and immune evasive Delta and Omicron variants [1]. Though there is evidence to support the view that booster doses following primary vaccine series induce neutralizing immunity against the Omicron variant [2], this variant is thought to be capable of disrupting herd immunity [3]. Until more robust, widely accepted vaccines are available, COVID-19 cases are likely to continue to rise, necessitating the development of additional treatment options [1]. Novel treatment strategies are urgently required to prevent severe disease, hospitalization and death, especially in vulnerable populations, such as the elderly and immunocompromised, as well as those with pre-existing conditions, including patients with cancer, or those who cannot get vaccinated. Here, we present integrin inhibition with or without MAP/ERK Kinase (MEK) inhibition as a potential treatment strategy for severe COVID-19.

Severe acute respiratory syndrome coronavirus 2 (SARS-CoV-2), the virus responsible for COVID-19, enters cells via interaction of its spike protein with angiotensin-converting enzyme 2 (ACE2) and possibly other receptors, such as CD147/26, on human cells [4,5,6,7]. The receptor-binding domain (RBD) of the spike contains a novel RGD (Arg-Gly-Asp) motif upstream from the ACE2 binding site that is absent in SARS-1 [8]. Of note, the Delta variant has two mutations in the RBD, while the Omicron variant has ten mutations [1]. The RGD motif was originally identified within several extracellular matrix proteins as the minimal peptide sequence required for cell attachment via integrins [9,10,11], which make transmembrane connections to the cytoskeleton and activate many intracellular signaling pathways [12]. Integrins are also commonly used as receptors by many human viruses [13]. The conservation of the motif and its localization in the receptor-binding region of the SARS-CoV-2 spike protein suggests that integrins may serve as alternative receptors for this virus [14]. Indeed, recent evidence suggests that this motif allows SARS-CoV-2 binding to integrins on human cells [15], facilitating viral infection [16], which may contribute to the higher transmission efficiency compared to SARS-CoV-1. There are eight known RGD-binding integrins with potential to impact on the pathogenesis of SARS-CoV-2: αvβ1, αvβ3, αvβ5, αvβ6, αvβ8, α5β1, α8β1, and αIIbβ3 [8]. A recent study has shown that blocking integrin αVβ3 prevents SARS-CoV-2 from binding to the vascular endothelium, potentially inhibiting virus-induced loss of endothelial barrier integrity and spread of SARS-CoV-2 to other organs [17]. Recent evidence supports the fundamental role of endothelial dysfunction in the systemic manifestations of COVID-19 [18,19]. Indeed, serum endothelial cell adhesion molecules are elevated in COVID-19 patients [20], and therapeutic intervention to improve endothelial dysfunction may decrease the length of hospitalization and reduce the need for respiratory support [21]. Other studies have shown that ATN-161, an integrin-binding peptide, is able to inhibit the binding of SARS-CoV-2 spike protein to integrin α5β1, resulting in diminished SARS-CoV-2 infection in vitro [22]. Furthermore, integrin α5β1 was targeted in vivo using ATN-161 and showed promising therapeutic efficacy [23]. Thus, therapeutic inhibition of RGD-binding integrins may provide benefit to COVID-19 patients.

We previously demonstrated that treatment of normal human small airway epithelial (HSAE) cells with various MEK inhibitor (MEKi) compounds, such as VS-6766, reduced cellular expression of ACE2 and inhibited pseudovirus infection [24]. Thus, we hypothesized that GLPG-0187 and VS-6766 may have an additive or synergistic inhibitory effect on pseudovirus infection. VS-6766 received the FDA Breakthrough Therapy Designation in combination with defactinib for treatment of ovarian cancer in 2021 [25], easing potential clinical translation.

We previously reported that SARS-CoV-2 pathogenesis can lead to a myriad of changes in cytokine, chemokine, and growth factor profiles in patient plasma samples and that these changes are associated with disease severity [26]. We recognized in our previous study that COVID-19 disease severity was associated with macrophage activation syndrome. Integrins can activate transforming growth factor beta (TGF-β), a growth factor secreted as a latent complex, which plays a role in the immune response [27], fibrosis [28], and viral replication [29]. The TGF-β complex consists of three proteins, including TGF-β, latency-associated protein (LAP), and an extracellular matrix-binding protein. LAP contains an RGD integrin-binding site, which mediates the activation of latent TGF-β via RGD-binding integrins [30]. The chronic immune response observed with SARS-CoV-2 is believed to be mediated by TGF-β [31]. Thus, it has been suggested that SARS-CoV-2 pathogenesis could be controlled via modulation of TGF-β [32].

Our past work demonstrated the feasibility of a SARS-CoV-2 pseudovirus model system to evaluate the effects of drug treatment on viral infection [24]. Here, we show that the pan-integrin inhibitor GLPG-0187 inhibits infection of multiple pseudovirus variants in HSAE cells, including the highly transmissible Delta variant, which was the most prevalent strain as of August 2021 [33], and the even more transmissible [2,3] Omicron variant, which became the most prevalent in December 2021 [34]. This finding is clinically relevant, as GLPG-0187 is in Phase I for treatment of solid tumors [35] and has shown a favorable toxicity profile in patients [36]. GLPG-0187 targets the integrins αvβ1, αvβ3, αvβ5, αvβ6, α5β1, and αvβ8, which, in addition to allowing infection of the virus, may play a potential role in SARS-CoV-2 pathogenesis by mediating activation of TGF-β, angiogenesis, lung injury, and inflammation [8,36].

Our current study suggests that Omicron may be less likely to infect lower airway cells in the lung compared to other COVID-19 variants and that integrin inhibitors have the potential to prevent infection with SARS-CoV-2, including the Delta and Omicron variants. This may decrease TGF-β levels, resulting in a decrease in COVID-19 severity, hospitalization, and death, especially in vulnerable and unvaccinated populations.

## 2. Results

### 2.1. Integrin Inhibition Decreases Infection of SARS-CoV-2 Pseudovirus Variants in Human Small Airway Epithelial Cells

**Figure 1:**
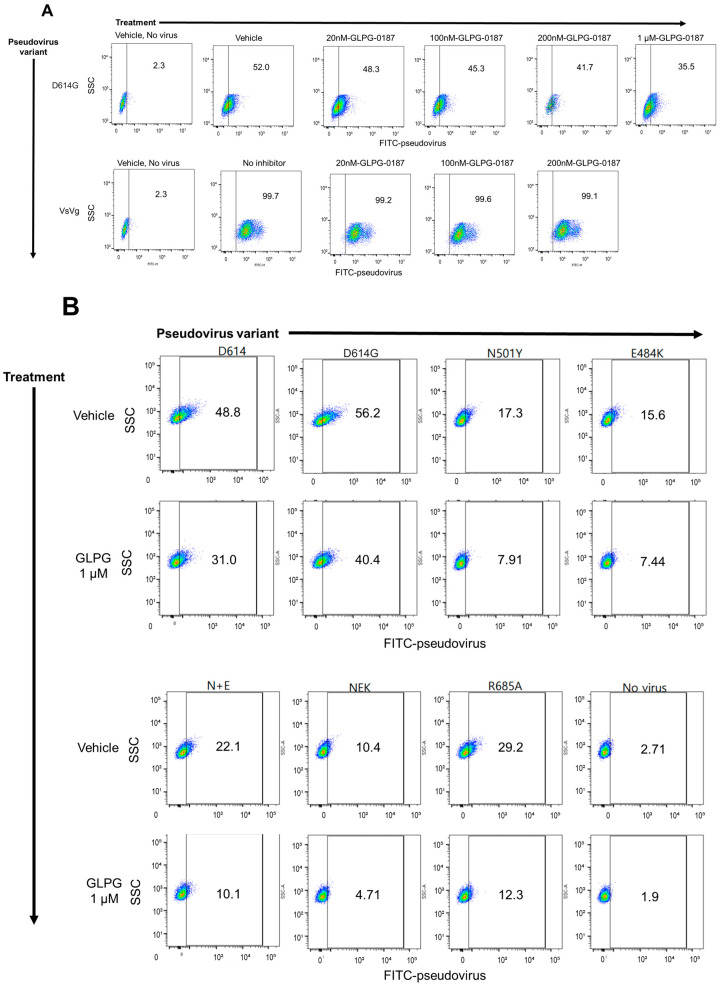

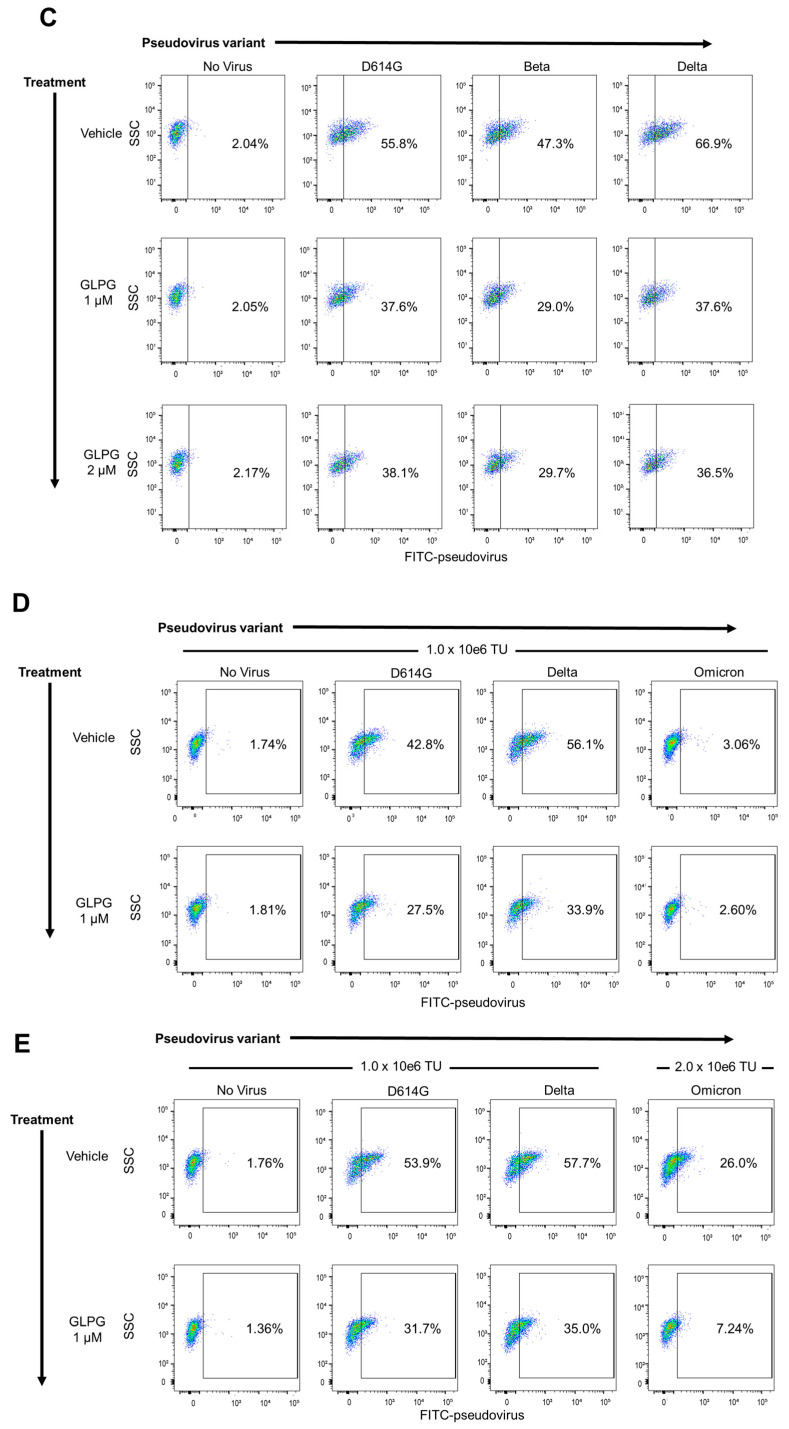
GLPG-0187 inhibits infection of SARS-CoV-2 pseudovirus variants D614, D614G, N501Y, E484K, N + E, NEK, R685A, Beta, Delta, and Omicron in small airway epithelial cells. (**A**) Treatment with 20 nM, 100 nM, 200 nM, or 1 µM GLPG-0187 for 2 h inhibits infection by the D614G pseudovirus variant (24 h infection time) in small airway epithelial cells compared to the VsVg positive control in a dose-dependent manner. DMSO was used as a vehicle control. (**B**) Treatment with 1 µM GLPG-0187 for 3 h inhibits infection by the D614, D614G, N501Y, E484K, N + E, NEK, R685A pseudovirus variants (20 h infection time). (**C**) Treatment with 1 µM or 2 µM GLPG-0187 for 2 h inhibits infection by the D614G, Beta, and Delta pseudovirus variants (20 h infection time). (**D**) Differential rates of infectivity across D614G, Delta, and Omicron variants observed after cells were spin-infected with the same amount of pseudovirus particles (1.0 × 10^6^ transduction units (TU) per 1 × 10^5^ cells/well) using the same experimental conditions described in panel C. (**E**) Treatment with 1 µM GLPG-0187 for 2 h inhibits infection by the Omicron pseudovirus variant (26 h infection time).

To test the inhibition of SARS-CoV-2 pseudovirus infection with the integrin inhibitor GLPG-0187, HSAE cells were pre-treated with 20 nM, 100 nM, 200 nM, or 1 µM GLPG-0187 for 2 h followed by spin infection with either a pseudovirus expressing the D614G spike protein variant or a VsVg positive control for 24 h. Few ZsGreen+ cells were seen in the cells not treated with spin infection, efficient viral infection was observed in cells treated with spin infection, and no effect of the inhibitor was observed on the VsVg positive control, as expected. Treatment with GLPG-0187 inhibited pseudovirus infection in a dose-dependent manner in the D614G variant (Figure 1A). In addition to D614G, several other SARS-CoV-2 pseudovirus variants were also tested, including D614, N501Y, E484K, N501Y + E484K (N + E), N501Y + E484K + K417N (NEK), R685A. Descriptions of these variants can be found in Table 1. HSAE cells pre-treated with 1 µM GLPG-0187 for 3 h followed by spin infection with pseudovirus variants for 20 h demonstrated inhibition of viral infection of each variant (Figure 1B). To test the inhibition of Beta and Delta variant pseudovirus infection, HSAE cells were pre-treated with 1 or 2 µM GLPG-0187 for 2 h followed by spin infection with pseudovirus. Pre-treatment with the integrin inhibitor resulted in the most significant decrease in pseudovirus infection by the Delta variant (Figure 1C). We conducted experiments with Omicron pseudovirus infection on HSAE cells with or without the integrin inhibitor GLPG-0187 (Figure 1D,E). The results suggest that the Omicron pseudovirus was less capable of infecting small airway epithelial cells than the D614G or Delta variant pseudovirus, which is in agreement with a recent study by Meng et al. [37]. Nevertheless, the integrin inhibitor GLPG-0187 effectively partially blocked D614G, Delta, and Omicron pseudovirus infection of HSAE cells.

### 2.2. MEK Inhibitor Pre-Treatment Enhances Inhibition of Pseudovirus Infection by GLPG-0187 in Human Small Airway Epithelial Cells

**Figure 2:**
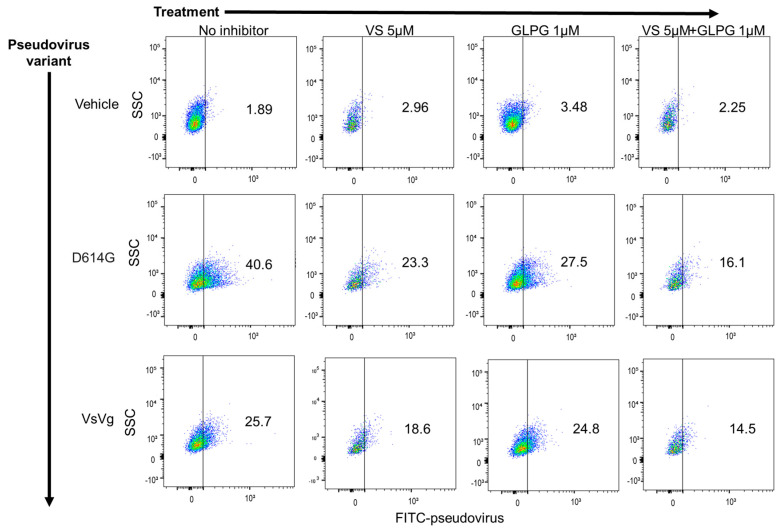
MEK inhibitor VS-6766 enhances the inhibition of SARS-CoV-2 pseudovirus infection by integrin inhibitor GLPG-0187 in small airway epithelial cells. Treatment with 5 µM VS-6766 for 24 h or with 1 µM GLPG-0187 for 3 h inhibits infection by the D614G pseudovirus variant (20 h infection time) in small airway epithelial cells compared to the VsVg positive control. DMSO was used as a vehicle control. Combination treatment involved 24 h pre-treatment with VS-6766 followed by an additional 3 h of treatment with GLPG-0187. The y axis shows side scatter, and the x axis shows FITC-pseudovirus expression.

We have previously demonstrated that MEKi compounds, including VS-6766, reduce cellular expression of ACE2 and inhibit pseudovirus infection of multiple human cell types [24]. Thus, we hypothesized that VS-6766 and GLPG-0187 could have an additive or synergistic inhibitory effect on pseudovirus infection of lung epithelial cells. To investigate this, we pre-treated HSAE cells with either 5 µM VS-6766 for 24 h, 1 µM GLPG-0187 for 3 h, or 5 µM VS-6766 for 24 h followed by an additional 3 h with GLPG-0187. After drug treatment, cells were spin-infected with the D614G pseudovirus for 20 h. As expected, VS-6766 and GLPG-0187 single treatment inhibited pseudovirus infection when compared to the positive control. A combination treatment enhanced the inhibition of pseudovirus infection compared to single agent treatment with either VS-6766 or GLPG-0187 (Figure 2). MEKi treatment also seemed to inhibit entry of the VsVg positive control pseudovirus, possibly due to unidentified off-target effects. Despite this, inhibition of the SARS-CoV-2 pseudovirus entry by MEKi treatment was enhanced compared to the positive control.

### 2.3. Plasma TGF-β1 Levels Correlate with Age, Race, and Number of Medications Administered upon Presentation with COVID, but Not with Sex

**Figure 3:**
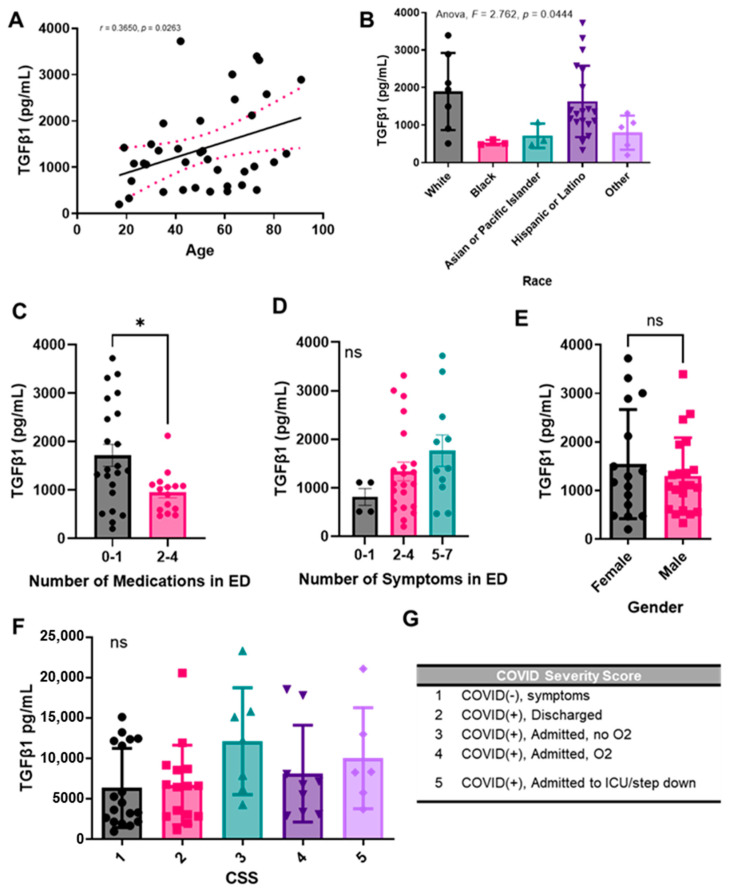
Plasma TGF-β1 levels correlate with age, race, and number of medications administered upon presentation with COVID-19 to the ED, but not with sex. Total TGF-β1 levels were detected in activated plasma samples. TGF-β1 plasma concentration correlation with (**A**) age, (**B**) race, (**C**) number of medications administered upon presentation with COVID-19 to the emergency department (ED), (**D**) number of symptoms reported upon presentation to the ED, (**E**) sex, or (**G**) COVID-19 severity score. (**G**) COVID-19 severity score (CSS) legend. Sample values are reported in pg/mL (*n* = 81 samples). Statistical significance was calculated using: (**A**) Spearman’s correlation, (**B**,**D**,**F**) One-way Anova followed by a post hoc Tukey’s multiple comparisons test, and (**C**,**E**) two-tailed, unpaired Student’s *t*-test. The minimal level of significance was *p* < 0.05. Bar graphs represent the mean of the population, and error bars indicate standard deviation. * represents *p* < 0.05.

Because it has been previously shown that the chronic immune response observed with SARS-CoV-2 is mediated by TGF-β, we sought to compare the levels of TGF-β1 in plasma samples from COVID-19 (+) patients upon admission to the emergency department (ED). We chose to focus on TGF-β1, as opposed to TGF-β2 and 3, since it has been previously shown that SARS-CoV-2 infection increased TGF-β1 expression in human epithelial cells and it is a known driver of lung fibrosis [38]. We analyzed the levels of total TGF-β1 in COVID-19 (+) plasma samples and found a significant correlation between TGF-β1 concentration (pg/mL) and age (Figure 3A). We also found significant variations in TGF-β1 concentrations depending on the patient’s self-reported race or ethnicity, with notably higher levels of the growth factor in White and Hispanic or Latino populations, and notably lower levels in Black and Asian or Pacific Islander populations (Figure 3B). We next grouped patients by the number of medications they received upon disease presentation to the ED (Figure 3C). Medications reported included ibuprofen, acetaminophen, bronchodilators (e.g., Albuterol), steroids (e.g., Prednisone), azithromycin, hydroxychloroquine, antibiotic, or other. We noticed statistically significant decreased plasma TGF-β1 concentrations in patients who received 2–4 medications in the ED, as compared to patients who received 0–1. Next, we grouped patients by number of symptoms self-reported upon admission to the ED and noted a positive trend between TGF-β1 levels and the number of symptoms, although not significant (Figure 3D). When comparing TGF-β1 levels between males and females, we did not note a significant difference (Figure 3E).

We also compared TGF-β1 levels in patients based on our COVID-19 Severity Score (CSS) (Figure 3F), which was based on the presence or absence of symptoms, patient oxygen requirements, and whether or not the patient was admitted to the ICU/step down units (Figure 3G). We again noted a positive trend between growth factor levels and increasing COVID-19 severity. Because we were interested in the role of TGF-β1 in the pathogenesis of other diseases as well, we also compared TGF-β1 levels in patients with a prior history of disease, including chronic lung disease, chronic kidney disease, chronic heart disease, pneumonia, high blood pressure, diabetes, previous stroke, and abnormal chest X-ray upon ED admission (Supplementary Figure S1). However, due to a limited sample size, we only noted a significant increase in TGF-β1 in patients with a history of chronic kidney disease as compared to those without a history of chronic kidney disease.

### 2.4. Active Plasma TGF-β1 Levels Correlate with Total TGF-β1 Levels

**Figure 4:**
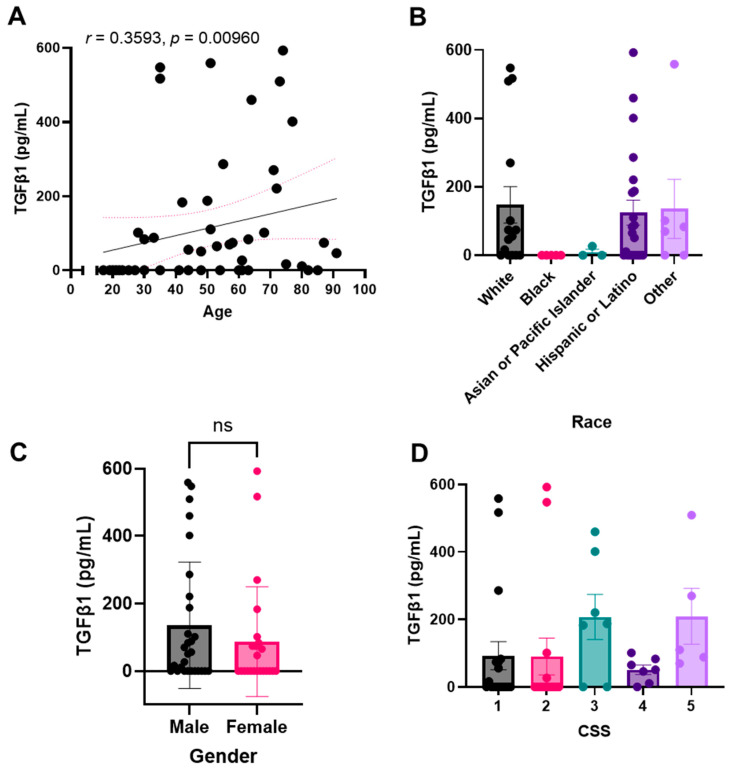
Active plasma TGF-β1 levels correlate with total TGF-β1 levels. Active TGF-β1 levels were detected in non-activated plasma samples. TGF-β1 plasma concentration correlation with (**A**) age, (**B**) race, (**C**) sex, or (**D**) COVID-19 severity score (CSS). Sample values are reported in pg/mL (*n* = 81 samples). Statistical significance was calculated using: (**A**) Spearman’s correlation, (**B**,**D**) One-way Anova followed by a post hoc Tukey’s multiple comparisons test, and (**C**) two-tailed, unpaired Student’s t-test. Bar graphs represent the mean of the population, and error bars indicate standard deviation. The minimal level of significance was *p* < 0.05.

Because we were interested in the concentrations of both active TGF-β1 and total TGF-β1, we next analyzed the patient plasma samples for active TGF-β1. We observed similar trends as described above and noted that active plasma TGF-β1 levels correlate with total TGF-β1 levels (Figure 4). We again noted a significant correlation between TGF-β1 plasma concentration and patient age (Figure 4A). We similarly noted higher levels of the growth factor in self-reported White and Hispanic or Latino populations and notably lower levels in Black and Asian or Pacific Islander populations (Figure 4B). When comparing active TGF-β1 levels between the sexes, we again did not note a significant difference (Figure 4C). Finally, we again noted a positive trend between active TGF-β levels and increasing COVID-19 severity, as determined by our CSS criteria (Figure 4D).

## 3. Discussion

SARS-CoV-2 remains a significant challenge in global health, and new treatment options are needed, especially for vulnerable and unvaccinated populations. As of November 2021, the Delta variant accounted for more than 99% of COVID-19 cases, and infection with this variant may result in an increased likelihood of hospitalization [39]. Since then, the Omicron variant rapidly spread around the globe and became the dominant strain in many parts of the world [34]. 

Our current study suggests that integrin inhibition reduces infection of multiple SARS-CoV-2 pseudovirus variants in HSAE cells and that GLPG-0187 may be particularly effective in inhibiting infection by the Delta variant. These effects were seen at short time points of 2–3 h, and future work could involve the evaluation of later time points to enhance inhibition of pseudovirus entry. As HSAE cells are thought to have very low expression of ACE2, our data suggest that alternative targets, such as RGD-binding integrins, may have particular value for treatment of COVID-19 [24,40,41]. Our findings also suggest that a combination treatment with a MEKi enhances inhibition of pseudovirus entry. In addition to inhibiting viral infection, it is possible that integrin inhibition could provide benefit to COVID-19 patients by reducing levels of active TGF-β, as integrins are a major regulator of TGF-β activation [42]. Limited prior studies have reported that COVID-19 patients may have higher levels of TGF-β compared to healthy controls, which may mediate some of the complications in severe COVID-19 patients [8]. Others have reported that GLPG-0187 and other integrin inhibitors decrease cellular TGF-β signaling [43,44]. Thus, treatment with GLPG-0187 may especially benefit populations of patients with high levels of TGF-β1. Integrin inhibition may provide benefit to COVID-19 patient populations with particularly high levels of TGF-β, such as elderly, White and Hispanic or Latino patients, and patients who receive few medications in the ED, report a high number of symptoms, have a high CSS, and/or have a history of chronic kidney disease. We also observed that TGF-β1 levels were lower in patients who identified as Black, despite recent reports that this population is more severely impacted by SARS-CoV-2 [45]. We hypothesize that variables such as living condition, social environment, and work situation may play important roles in this result. Moreover, we noted that there were no statistically significant differences in TGF-β1 levels based on sex, which is a reported risk factor, and hypothesize that a larger sample size or samples taken at different time points during infection may lead to sex-based differences in TGF-β1 levels.

Repeat experiments, including those conducted with authentic SARS-CoV-2 virus, are needed prior to clinical translation of these treatments. Other limitations of this study include the small sample size of plasma samples from patients with COVID-19, as well as the lack of serial samples over time from the same patient. Because we only analyzed plasma from patients upon admission to the ED, we may have missed fluctuations in TGF-β concentrations, which are thought to peak during the first two weeks post-infection in severe COVID-19 cases [46]. Future work should monitor TGF-β dynamics in serial patient samples, as well as in various cell culture supernatant samples post-treatment with GLPG-0187. It should also be noted that a small proportion of latent TGF-β may have been activated by freezing and thawing of the samples, which could contribute to the relationship between active and total TGF-β that we observed.

Since its first identification in South Africa in November 2021, the SARS-CoV-2 Omicron variant raised serious concerns of a significant reduction in efficacy of vaccines and monoclonal antibody treatments and an increased risk of reinfection due to numerous mutations in its spike protein, which is the antigenic target of infection- and vaccine-elicited antibodies against SARS-CoV-2. Currently, the Omicron variant is on track to outcompete the Delta variant, as cases have soared to record highs in parts of Europe and now the U.S. according to the data released by Johns Hopkins University [47]. A number of recent studies suggest that much of the Omicron variant’s dominance comes down to its ability to evade the body’s immune defenses [37,48,49,50,51]. However, earlier analyses of patients in South Africa suggest Omicron-infected individuals had a reduced risk of severe disease when compared to Delta-infected individuals [50]. In the first findings on how the Omicron variant infects the respiratory tract, researchers from Hong Kong University reported that the virus multiplies 70 times faster in the bronchi than Delta and the original SARS-CoV-2 virus [34]. In a potential clue regarding lower disease severity, they found that Omicron replication was less efficient in deeper lung tissue, more than ten times lower than the original virus. In a recent study by Meng et al. [37], the investigators found that despite three mutations predicted to favor spike S1/S2 cleavage, the observed cleavage efficiency was substantially lower than for Delta, and Omicron pseudovirus entry into lower airway organoids and Calu-3 lung cells was thus impaired. In the latest study on mice and hamsters [49], Omicron produced less-damaging infections, often limited largely to the upper airway, including the nose, throat, and windpipe. The variant did much less harm to the lungs, whereas previous variants would often cause scarring and serious breathing difficulty. In our current study, we found that the Omicron pseudovirus was less capable of infecting the small airway epithelial cells than D614G or Delta variant pseudovirus and that integrin inhibition effectively blocked D614G, Delta, and Omicron pseudovirus infection of the small airway epithelial cells. Combined, these observations highlight that Omicron has gained immune evasion properties while compromising cell entry in lung cells, with possible implications for altered pathogenicity. In addition, targeting alternative viral infection routes, such as integrin-mediated cell entry, and dampening TGF-β1-mediated disease severity may have therapeutic implications, especially for vulnerable and unvaccinated populations.

It is possible that GLPG-0187 inhibits pseudovirus variant infection by an off-target effect on ACE2. It is also possible that (1) the virus may infect ACE2 negative cells by using RGD-binding integrins as an alternative receptor to ACE2 and/or (2) the RGD motif functions as a co-receptor that enhances viral infection via ACE2. Future work should involve similar experiments in cells that are completely ACE2 negative, either naturally or by genetic modification, as low levels of ACE2 expression may still be relevant for viral infection. Moreover, because it has been recently demonstrated that the Omicron variant shows rapid replication in nasal epithelial cells, similar experiments may be conducted in this cell type to determine the inhibition of pseudovirus entry post-treatment with GLPG-1087 [52]. Future work may also include the development of a pseudovirus with a mutated RGD motif to assess the effects on viral infection, as well as to analyze which RGD-integrin(s) are important for SARS-CoV-2 infection. Our results, nonetheless, demonstrate that GLPG-0187 inhibits pseudovirus entry, providing rationale for further investigation of integrin inhibitors and other host cell targeted therapies as a potential therapy for COVID-19 [53,54,55]. Moreover, our findings offer a combinatorial strategy, combining an integrin inhibitor with a MEK inhibitor as a therapeutic strategy against COVID-19, including Delta and Omicron variants. These strategies could be further tested in clinical trials with particularly at-risk patients with COVID-19 infection who are unvaccinated, immunosuppressed, or with risk factors such as comorbidities, including cancer.

## 4. Methods

### 4.1. Cell Culture

HSAE cells (ATCC PCS-301-010) were cultured in Airway Epithelial Cell Basal Medium (ATCC PCS-300-030) supplemented with the Bronchial Epithelial Cell Growth Kit (ATCC PCS-300-040) at 37 °C in humidified atmosphere containing 5% CO_2_.

### 4.2. SARS-CoV-2 Pseudoviruses and Cell Entry Assays

We developed a SARS-CoV-2 pseudovirus model system that uses pseudotyped SARS-CoV-2 viruses with a lentiviral core and a variety of SARS-CoV-2 spike protein variants on its envelope. To assess the infectivity of normal human small airway epithelial (HSAE) cells, we used flow cytometry to quantify infected cells that express a fluorescence protein ZsGreen, which was detected using the FITC channel. A replication-incompetent SARS-CoV-2 pseudovirus was generated using a lentiviral packaging system as previously described [24]. Briefly, 293FT cells (Invitrogen) at 75% confluency were co-transfected with the backbone vector pHAGE-fullEF1α-Luciferase-IRES-ZsGreen plasmids expressing lentiviral proteins Tat, Rev and Gag/Pol, and plasmids expressing D614 or D614G S protein (a gift from Dr. Hyeryun Choe, The Scripps Research Institute, Jupiter, FL, USA), or S protein with N501Y, E484K, N501Y + E484K or N501Y + E484K + K417N mutations. An S protein expression plasmid construct containing all Beta variant (B.1.351) mutations and another S protein construct containing all Delta variant (B.1.617.2) mutations were gifts from Drs. Markus Hoffmann and Stefan Poehlmann [56] (German Primate Center, Goettingen, Germany). An S protein expression plasmid construct containing all Omicron variant (B.1.1.529) mutations [48] was custom-made by GenScript (Piscataway, NJ, USA): pcDNA3.1(+)-SARS-CoV-2-Omicron-(6xHis)-Spike (human codon). The variant S genes in the above constructs were sequenced to confirm all the corresponding mutations (Table 1). A plasmid expressing VsVg protein instead of the S protein was used to generate a pantropic control lentivirus. Cell culture supernatants were collected, filtered, concentrated using ultra-centrifugation, aliquoted, and frozen at −80 °C. Virus titer was determined using Lenti-X™ p24 Rapid Titration ELISA Kit (Takara Bio Inc., Shiga, Japan), and lentiviral particles were analyzed on an SDS-PAGE gel followed by Western blot to detect C-terminal FLAG-tagged S protein. HSAE cells were pre-treated with the integrin inhibitor GLPG-0187 (Galapagos NV, Mechelen, Belgium), MEK inhibitor VS-6766 (Verastem Oncology, Needham, MA, USA), or both, for 2–27 h. Following drug treatment, HSAE cells were spin-infected with SARS-CoV-2 pseudoviruses or a pantropic VsVg positive control lentivirus in a 12-well plate (931 g, 2 h, 30 °C with 8 μg/mL polybrene). Analysis of ZsGreen+ cells was conducted by flow cytometry 20–24 h after infection using a BD LSRII flow cytometer and FlowJo software (FlowJo, LLC, Ashland, OR, USA). DAPI was used to exclude dead cells. ZsGreen+ cells were gated on based on an unstained, uninfected HSAE cell control, as previously described [24].

### 4.3. Human Plasma Samples

COVID-19 (+) human plasma samples were received from the Lifespan Brown COVID-19 Biobank at Rhode Island Hospital (Providence, RI, USA). All patient samples were deidentified but contained associated clinical information, as described. The IRB study protocol “Pilot Study Evaluating Cytokine Profiles in COVID-19 Patient Samples” did not meet the definition of human subject research by either the Brown University or the Rhode Island Hospital IRBs.

IRB/oversight of exemption for the research (as previously described) [26].

COVID-19 (+) and (−) human plasma samples were received from the Lifespan Brown COVID-19 Biobank from Brown University at Rhode Island Hospital (Providence, Rhode Island). All patient samples were deidentified but included the available clinical information as described. The IRB study protocol “Pilot Study Evaluating Cytokine Profiles in COVID-19 Patient Samples” did not meet the definition of human subject research by either the Brown University or the Rhode Island Hospital IRBs. This is based on the fact that the project used deidentified specimens from a biobank with a determination that this project did not meet the definition of human subject research based on specific criteria as described below. The original samples were collected at Rhode Island hospital by the Lifespan Brown COVID-19 Biobank through an IRB-approved protocol that involved informed consent, which was used by the biobank. We completed a human subjects determination form for the Human Subjects Protection Program at Brown University. We explained the purpose of our research and that we would be receiving deidentified samples from the COVID-19 biobank. We further answered questions about our study that led to the determination that our study constitutes research because we answered “yes” to the following two questions: (1) Does your proposed project involve a systematic investigation, that is, a prospective plan that incorporates qualitative or quantitative data collection and data analysis to answer a question; and (2) Is the intent of your proposed project to develop or contribute to generalizable knowledge, that is, to create knowledge from which conclusions will be drawn that can be applied to populations beyond the specific population from which it was collected. In addition, we answered “no” to four questions regarding whether the project involves human subjects. The questions were: (1) Does your proposed project involve an intervention, that is, a physical procedure or manipulation or a living individual (or their environment) to obtain information about them; (2) Does your proposed project involve an interaction, that is, communication or contact with a living individual (in person, online, or by phone) to obtain information about them; (3) Does your proposed project involve identifiable private information or identifiable biospecimen, that is, receipt or collection of private information or biospecimen about a living individual to obtain information about them; and (4) Does your proposed project involve coded information/biospecimens, that is, where a link exists that could allow information about a living individual to be reidentified AND you are able to access the link. Since we answered “no” to all these questions, our proposed project did NOT involve “Human Subjects.” Based on the information included in the Human Subjects Determination Form, The Human Research Protection Program at Brown University agreed with the investigator’s self-determination that the project does not meet the definition of human subject research. This determination was made by the Human Research Protection Program at Brown University on 17 June 2020.

### 4.4. Cytokine Profiling

A Human Magnetic Luminex Performance Assay TGF-β1 Base Kit (Cat # LTGM100, R&D Systems, Inc., Minneapolis, MN, USA) was run on a Luminex 200 Instrument (LX200-XPON-RUO, Luminex Corporation, Austin, TX, USA) according to the manufacturer’s instructions. Total TGF-β1 was quantified by activating patient samples with 1N HCl, neutralizing with 1.2N NaOH/0.5M HEPES, and then immediately assaying for TGF-β1. Active TGF-β1 was quantified without sample activation or neutralization prior to analysis.

### 4.5. Statistical Analysis

Spearman’s correlation was used to calculate statistical significance of the scatter plots, while the statistical significance between groups was determined using a one-way Anova followed by a post hoc Tukey’s multiple comparisons test. A two-tailed, unpaired Student’s *t*-test was used to calculate the statistical significance of pairs. The minimal level of significance was *p* < 0.05. The following symbols, * and **, represent *p* < 0.05 and *p* < 0.01, respectively.

## 5. Conclusions

Here, we show that pan-integrin inhibitor GLPG-0187 reduces SARS-CoV-2 pseudovirus infection of HSAE cells. Omicron pseudovirus infectivity was significantly reduced as compared to D614G or Delta variants, and GLPG-0187 reduced SARS-CoV-2 pseudovirus infection in a dose-dependent manner across multiple viral variants. This inhibition was most efficient in the Omicron and Delta variants. VS-6766 enhanced inhibition of pseudovirus infection by GLPG-0187. We compared plasma levels of active and total TGF-β in COVID-19+ patients because integrins activate TGF-β signaling. Plasma TGF-β1 levels correlated with age, race, and number of medications upon presentation with COVID-19, but not with sex. Total plasma TGF-β1 levels correlated with activated TGF-β1 levels. Integrin inhibition as a therapeutic strategy may be further explored through additional preclinical and clinical testing in vulnerable and unvaccinated populations.

## Figures and Tables

**Table 1:** Description of several SARS-CoV-2 viral spike variants that are represented in this study with experimental pseudoviruses.

Variant	Description
Omicron (B.1.1.529)	Dominant strain as of December 2021. Spike mutations include: A67V, Δ69–70, T95I, G142D, Δ143–145, Δ211, L212I, ins214EPE, G339D, S371L, S373P, S375F, K417N, N440K, G446S, S477N, T478K, E484A, Q493R, G496S, Q498R, N501Y, Y505H, T547K, D614G, H655Y, N679K, P681H, N764K, D796Y, N856K, Q954H, N969K, L981F
Delta (B.1.617.2)	Dominant strain as of August 2021. Spike mutations include: T19R, G142D, E156G, F157Δ, R158Δ, L452R, T478K. D614G, P681R, D950N
Beta (B.1.351)	Prevalent in late 2020. Spike mutations include: L18F, D80A, D215G, Δ242–244, R246I, K417N, E484K, N501Y, D614G, A701V
D614G	Dominant strain in the spring of 2020
D614	Prevalent strain in early 2020
N501Y	A common mutation in the Alpha (B.1.1.7), Beta (B.1.351), and Gamma (P.1) variants
E484K	A common mutation in the Beta (B.1.351) and Gamma (P.1) variants
N + E (N501Y + E484K)	Common mutations in the Beta (B.1.351) and Gamma (P.1) variants
NEK (N501Y + E484K + K417N)	Common mutations in the Beta (B.1.351) and Gamma (P.1) variants
R785A	Furin-cleavage site mutated

## Data Availability

Data is contained within the article.

## Supplementary Materials

The following supporting information can be downloaded at: https://www.mdpi.com/article/10.3390/ph15050618/s1, Figure S1. Plasma TGF-β1 levels are elevated in patients with a history of kidney disease. Total TGF-β1 levels were detected in activated plasma samples. TGF-β1 plasma concentration correlated with (A) history of chronic lung disease, (B) history of chronic kidney disease, (C) history of chronic heart disease (D) pneumonia upon presentation to the emergency department (ED), (E) history of high blood pressure, (F) history of diabetes, (G) history of stroke, or (H) chest x-ray upon presentation to the ED. Statistical significance was calculated using a two-tailed, unpaired Student’s *t*-test. The minimal level of significance was *p* < 0.05 indicated by *. Bar graphs represent the mean of the population, and error bars indicate the standard deviation.

## Abbreviations

ACE2	angiotensin-converting enzyme 2
COVID-19	coronavirus disease 2019
CSS	COVID-19 severity score
ED	emergency department
HSAE	human small airway epithelial
LAP	latency-associated protein
MEK	MAP/ERK kinase
MEKi	MEK inhibitor
RBD	receptor-binding domain
SARS-CoV-2	severe acute respiratory syndrome coronavirus 2
TGF-β	transforming growth factor beta
TU	transduction units
