# Supplementary material for: Integrin/TGF-β1 Inhibitor GLPG-0187 Blocks SARS-CoV-2 Delta and Omicron Pseudovirus Infection of Airway Epithelial Cells In Vitro, Which Could Attenuate Disease Severity
Source: Pharmaceuticals (Basel). 2022 May 17;15(5):618. doi: 10.3390/ph15050618 (PMC9143518; doi:10.3390/ph15050618)
Supplement: Supplementary file 1 [file pharmaceuticals-15-00618-s001.zip › pharmaceuticals-1648012-supplementary.pdf]

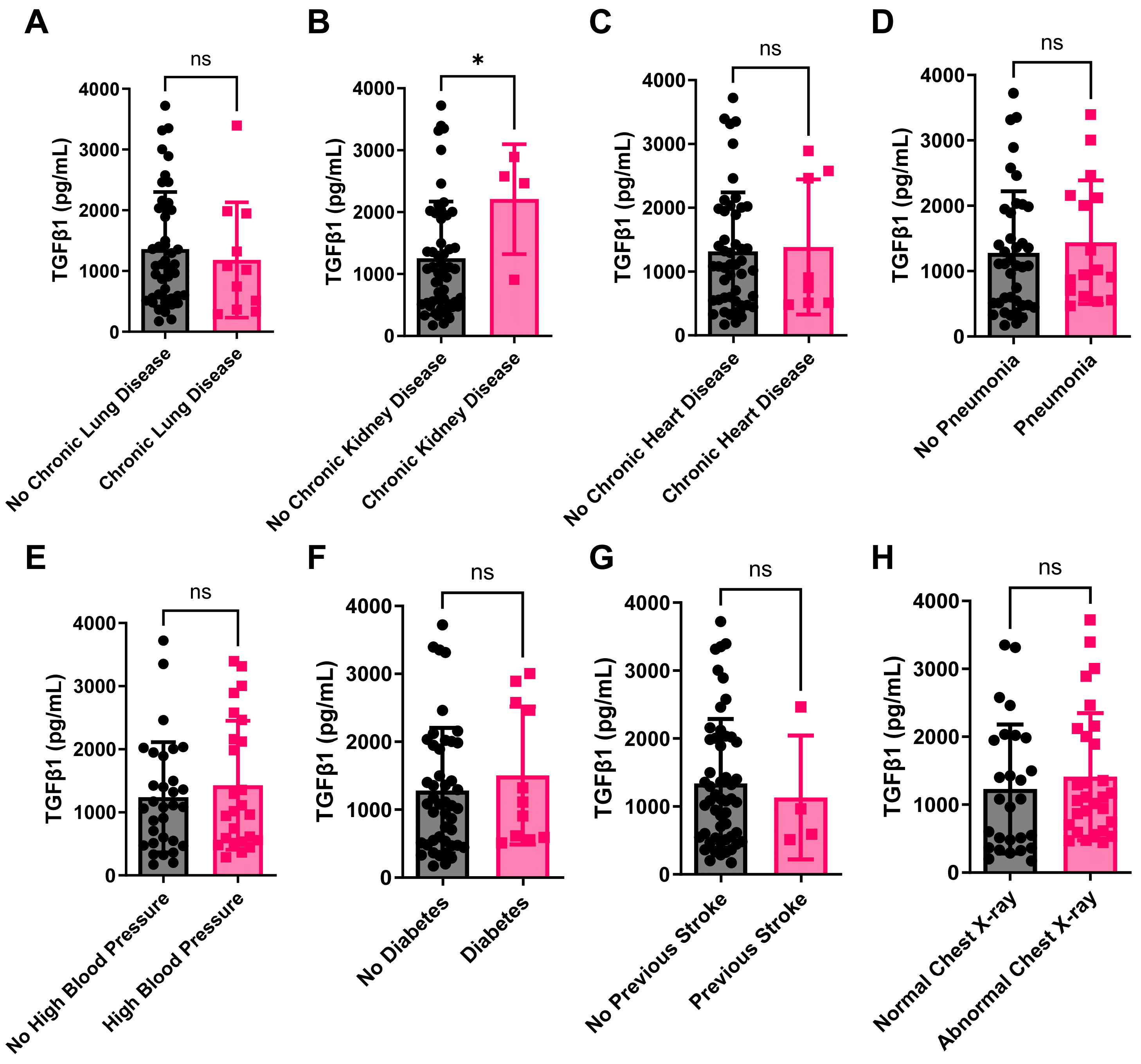

Supplementary Figure S1. Plasma TGF-β1 levels are elevated in patients with a history of kidney disease. Total TGF-β1 levels were detected in activated plasma samples. TGF-β1 plasma concentration correlated with (A) history of chronic lung disease, (B) history of chronic kidney disease, (C) history of chronic heart disease (D) pneumonia upon presentation to the emergency department (ED), (E) history of high blood pressure, (F) history of diabetes, (G) history of stroke, or (H) chest x-ray upon presentation to the ED. Statistical significance was calculated using a two-tailed, unpaired Student's t-test. The minimal level of significance was $p < 0.05$ indicated by *. Bar graphs represent the mean of the population, and error bars indicate the standard deviation.
